# The Effect of Armed Conflict on the Utilization of Maternal Health Services in Uganda: A Population-based Study

**DOI:** 10.1371/currents.dis.557b987d6519d8c7c96f2006ed3c271a

**Published:** 2017-10-03

**Authors:** Amrita Namasivayam, Pedro Arcos González, Rafael Castro Delgado, Primus Che Chi

**Affiliations:** School of Health Sciences, University of Canterbury, Christchurch, New Zealand; Unit for Research in Emergency and Disaster, Department of Medicine, University of Oviedo, Oviedo, Spain; Unit for Research in Emergency and Disaster, Department of Medicine, Oviedo University, Oviedo, Spain

## Abstract

**Introduction:**

Maternal mortality rates can be adversely affected by armed conflict, implying a greater level of vulnerability among women, and is often linked to the lack of or limited access to maternal healthcare during conflict. Previous research in Uganda has shown that armed conflict negatively impacts women’s utilization of maternal healthcare services for a multitude of reasons at the individual, health-system and political levels.

**Methods:**

This study compared aggregated Demographic and Health Surveys data from 13 districts in Northern Uganda, a conflict-affected region, with data from the rest of the country, for the use of maternal healthcare services for the years 1988, 1995, 2000, 2006 and 2011, using statistical analyses and logistic regression. Specific indicators for maternal healthcare utilization included contraceptive use, antenatal care, skilled assistance at birth and institutional delivery.

**Results:**

Use of contraception and institutional deliveries among women in Northern Uganda was significantly lower compared to the rest of the country. However, skilled assistance at birth among women in Northern Uganda was significantly higher.

**Conclusions:**

The findings in this study show that armed conflict can have a negative impact on aspects of maternal healthcare such as contraceptive use and institutional deliveries; however, other indicators such as skilled assistance at birth were seen to be better among conflict-affected populations. This reiterates the complex nature of armed conflict and the interplay of different factors such as conflict intensity, existing health systems and services, and humanitarian interventions that could influence maternal healthcare utilization.

**Key words:**

Armed conflict, maternal health utilization, Northern Uganda, contraception, skilled assistance at birth, antenatal care, institutional delivery

## Background

**Maternal health in context**

Current figures from the World Health Organization (WHO) on maternal health estimate that approximately 303,000 women die every year during pregnancy and childbirth; this amounts to a staggering 830 deaths every day^1^. An estimated 99% of all maternal deaths occur in low and middle income countries, with more than half of these deaths reported in sub-Saharan Africa (SSA) and another third in South Asia; together, these two regions account for 87% of all maternal deaths globally^2^^,^^3^.

Though addressing the problem of maternal mortality requires more than just a simple, one-size-fits-all approach 4, the provision of maternal healthcare services has repeatedly been shown to be essential in curbing maternal deaths^5^^,^^6^^,^^7^^,^^8^^,^^9^. These include access to antenatal care (ANC) during pregnancy^10^^,^^11^, skilled attendance at delivery^12^^,^^13^^,^^14^, deliveries at healthcare facilities or hospitals and access to emergency obstetric care^15^, and appropriate postpartum care^16^^,^^17^.

In 2015, the maternal mortality ratio in Uganda stood at 343 deaths per 100,000 live births^18^. While this figure has seen a decreasing trend over the years with significant improvements in maternal healthcare, it still remains very high as there continue to be gaps in the quality and accessibility of maternal healthcare services, a lack of trained staff, medicines and medical supplies. and inequities in service provision. Uganda faces a challenge in maternal mortality from causes related directly to pregnancy and childbirth, unsafe abortions and obstetric complications; this divide is worse among women in rural areas and those of lower socioeconomic status and education^19^.

**Armed conflict and its impact on maternal healthcare utilization**

The impact of conflict on the health and wellbeing of populations is caused by both the direct effects of combat- deaths and injuries sustained during battle- as well as from the more indirect consequences. The latter includes population displacement, the breakdown of social and health services^20^^,^^21^^,^^22^, and the increased risk of disease outbreaks due to poor shelter and sanitation, overcrowding and a lack of access to clean water and food. Armed conflict has also been shown to have a gendered effect on population health, where excess mortality attributed to conflict (both directly and indirectly) is higher among women than men, implying a greater level of vulnerability among women^23^^,^^24^. Maternal mortality in conflict can be particularly exacerbated by limited access to maternal health services due to safety, financial and geographical restrictions^25^, as well as the general collapse of the health system and disruption of routine health service delivery. The increased incidence of sexual violence and rape during conflict also increases rates of maternal morbidity and mortality^26^.

Uganda experienced a brutal civil war between the Lord’s Resistance Army (LRA) and the Government of Uganda for 20 years, between 1986 to 2006. Though the worst hit areas of the country were primarily in the northern regions (Gulu, Kitgum, Pader, Lira, Apac), the conflict affected many other areas due to the large numbers of internally displaced persons (IDPs) and the widespread terror and violence during the period of the 20 years^27^^,^^28^^,^^29^. In the course of the conflict, an estimated 500,000 people were killed and a further 2 million people internally displaced and approximately 66,000 children were kidnapped by the LRA. The economic and health status of these populations also deteriorated significantly during this time, increasing the levels of poverty and lack of education^28^.

**Rationale for the study**

Previous research has looked at maternal mortality and fertility rates during the conflict in Uganda, and qualitative studies have looked at the determinants, perceptions and barriers around maternal healthcare service utilization post conflict^25^^,^^26^^,^^30^. While these studies have predominantly been qualitative and based on interviews with respondents in a post-conflict setting, little has been published on the trends (and potential differences) in utilization of maternal health at the national level; both in regions of conflict compared to unaffected regions, and across the duration of the conflict period.

This study compared aggregated population-level data from the 13 districts in the Northern Uganda region, with data from the rest of the country, for statistically significant differences in the utilization of maternal healthcare services. The main hypothesis was that living in a conflict-affected region negatively impacts maternal healthcare utilization; the 5 time points of data served to provide a trend across the 20-year conflict period and beyond, to assess whether there were differences in maternal healthcare utilization during the conflict, compared to after, as well as at different time points during the conflict. While the first analysis looked directly at the geographical region (conflict versus non conflict) in the utilization of maternal health care, a second analysis adjusted for demographic factors such as age, education, parity, socioeconomic status and distance to the nearest health care service facility. These factors were selected based on previous research, showing their independent effects on maternal healthcare utilization^14^^,^^31^^,^^32^^,^^33^.

## Methodology

**Data source**

Demographic and Health Surveys (DHS) are nationally representative population-based surveys, commissioned by USAID and periodically carried out by the governments of different countries, with operational support from ICF International. Data sets are available through application to MEASURE DHS, and once a data request has been approved, no further ethical clearance is required for use of this data for research.

The DHS surveys employ stratified two-stage cluster design. Clusters are first selected from the most recent population census sample frame and households are subsequently and systematically selected from the clusters. Participants eligible for interview include all women aged 15–49 years and men aged 15–54 years who are residents of the selected household. The surveys use standardized questionnaires developed by the MEASURE DHS programme specifically for women, men and households; these are administered during face-to-face interviews. Detailed information about sampling methodologies and data collection procedures can be found in the DHS reports for respective countries^34^^,^^35^.

**Study sample**

Data for this study was obtained from the Uganda Standard data sets for the years 1988-89, 1995, 2000-2001, 2006, and 2011. Women’s individual datasets were used in the analysis; these consist of representative samples of women, married and unmarried, between the ages of 15-49, who were not pregnant at the time of the interview. Data from 4730 women in 1988/1989, 7070 women in 1995, 7246 women in 2000/2001, 8531 women in 2006 and 8674 women in 2011, were analyzed in this study.

**Variables and data analysis**

The variables used in this study are listed in Table 1 below. Data was analysed using Statistical Package for the Social Sciences (SPSS) version 23.0, with statistical significance set at p<0.05. Bivariate and multinomial logistic regression was used to determine if region (rest of the country vs. North) was significant in predicting differences in women’s utilization of maternal healthcare. Two levels of analyses were conducted; the first analysis was unadjusted and assessed only region as a predictor variable for women’s utilization of maternal healthcare, while the second analysis was adjusted by including age, education, parity, distance to the nearest health facility and wealth index of the respondents.

Measures of association are presented as odds ratios (ORs) and 95% confidence intervals (CIs). Odds above 1 signify a higher likelihood of utilizing maternal healthcare, and odds below 1 signify a lower likelihood of utilizing maternal healthcare. Missing data were excluded from the analysis.

**Figure:**
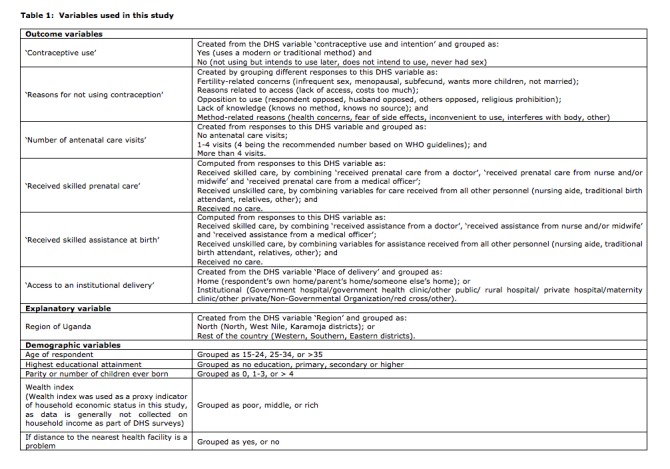
**Table 1:** Variables used in this study

## Results

Geographical region was included as the only predictor of maternal healthcare utilization in the unadjusted analysis to assess the independent effects of conflict before adjusting for other variables. Demographic characteristics were included in the adjusted analysis for any confounding or mediating effects that these variables may have on the association between region and maternal healthcare utilization (Table 2). The individual associations between the demographic variables and maternal healthcare utilization are not shown.

The odds for the use of contraception and institutional delivery for women in the north of Uganda were significantly lower compared to the rest of the country, across all 5 time points in the study. However, the odds for skilled assistance at birth were higher among women in the north of Uganda, across the conflict years 1988-2006; post conflict, this was no longer statistically significant. Odds for unskilled assistance at birth were significantly lower for the conflict years 1995 and 2006, and post-conflict.

With respect to the reasons for the non-use of contraception, the odds for fertility-related concerns, opposition to use, and lack of knowledge were significantly lower among women in the North across all time points in the study in both analyses, and in 2006 lack of access was significantly lower for women in the North as well.

Attendance at antenatal care sessions were not significant for the initial conflict years 1995 and 2001 in the unadjusted analysis; but in 2006 and post conflict, the odds for this indicator was significantly lower for women in the North. The odds for attendance at 4 or more sessions in the adjusted analysis were statistically significant and lower for women in the North in 1995 and 2000/2001 as well. The relative odds for unskilled assistance at prenatal care (from traditional birth attendants, relatives, etc.) was significantly low for some years, but not significant in others.

**Figure:**
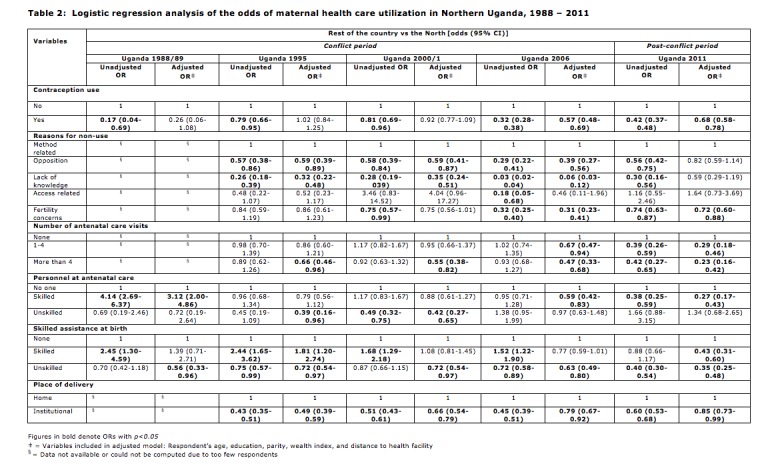
**Table 2:** Logistic regression analysis of the odds of maternal health care utilization in Northern Uganda, 1988-2011

## Discussion

The principal hypothesis of this study was that armed conflict negatively impacts maternal healthcare utilization. The findings of this study both support and contradict this to different extent and for different indicators, at different time points of the study period.

As the results for the use of contraception and an institutional delivery show, for women in the north of Uganda the odds are significantly lower for both indicators compared to the rest of the country, across all 5 time points in the study. This is as expected, given that armed conflict constrains availability of and access to healthcare due to security, geographical and financial reasons, as well as the reduced functionality of the health system^36^^,^^37^. Furthermore, previous studies have shown higher fertility rates among populations in conflict, citing this as a coping strategy where having a larger family provides more social and/or economic security, as well as families wanting to replace members lost in the conflict itself^25^. The lower use of contraception among conflict-affected populations therefore could reflect both affected weakened health system as well as a conscious attempt to preserve and possibly enlarge family size^38^. With regard to the reasons for non-use of contraception, the odds for opposition to use, fertility concerns and lack of knowledge were significantly lower among women in the North. These results too, could support the premise that family planning is not of top priority in times and regions of conflict.

The higher odds for skilled assistance at birth among women in the north of Uganda, across the years 1988-2006, could be indicative of interventions and/or humanitarian assistance specifically targeting women in this region to improve birth outcomes and delivery services through the training and deployment of skilled personnel such as midwives and obstetricians, regardless of whether women chose to or were able to deliver in the hospital or at home. As highlighted by Orach and De Brouwere^21^, camped populations in conflict such as IDPs and refugees can often have better access and options for health care services compared to host populations due to international aid and additional health facilities being established in these areas; this could lend explanation to the observation of improved use of skilled assistance at birth by women in conflict-affected areas. Interestingly, in 2011, skilled assistance at birth was no longer significantly higher, possibly implying the cessation of such healthcare interventions in the North in the post-conflict period, or the relocation of populations from camp settings back to their homes where access to healthcare and basic resources was once again poor or problematic^26^. Similarly, although attendance at antenatal care sessions were not significant for the years of conflict, in 2011 the odds for this indicator was significantly lower for women in the North, possibly again due to the withdrawal of humanitarian organizations providing maternal health services. Odds for unskilled assistance (from traditional birth attendants, relatives, etc.) was significantly low for some years, again possibly due to the availability of skilled assistance, or the difficulties and insecurities of reaching these persons in times of conflict^25^.

While an increase in maternal healthcare utilization might be expect over time (particularly in the post-conflict period, compared with the conflict period), this was not apparent in this study. One explanation could be that by 2011, the North had not yet recovered to an extent where full functionality of the healthcare system and services had been sufficiently restored to show an increased level of health service utilization^37^. Given the extent and long-term nature of the population displacements that resulted from the conflict, the return and resettlement of populations to their original homes and rebuilding of societies and services undoubtedly requires significant amounts of time and investment in the region^39^.

This study has several limitations. Given that it explores the topic of armed conflict using population data from over a period of more than 20 years, the quality and completeness of the data is a critical factor in analyzing trends in maternal health service utilization in the North compared to the rest of the country. The DHS data sets from 1988 and 2000/2001 excluded data from some Northern districts (Kitgum, Gulu, Apac, Lira, etc.) due to security issues with obtaining the data from these regions during the conflict, and hence there is the possibility of over-representation of data from the Northern districts that were not as badly affected by the conflict, and under-representation of the impact of the conflict in districts that were more seriously affected. Secondly, the indicators for maternal healthcare utilization used in this study were limited to those around contraception, antenatal care and delivery; many more indicators are available in the DHS datasets and could provide a more comprehensive understanding of other aspects of maternal healthcare utilization, such as postnatal care. DHS data from the 5 sample sets could also not be disaggregated beyond the regional level, thereby limiting the extent to which this data could be mapped and compared with conflict intensity data for this region, available through the Armed Conflict Location and Event Data Project^40^. This analysis, if it had been possible, would have provided a more comprehensive and in-depth look at the effect of conflict intensity on maternal health care utilization in the different districts in Northern Uganda, and a better sense of the correlation between the two variables. Thirdly, data from the partners of women in this study were not analyzed; this includes indicators such as partner’s education, control over earnings, decision-making autonomy in the household, and relationship inequality, among others. These factors have shown to have a possible impact on maternal healthcare utilization in different settings^41^, and for future work, could provide an added level of analysis specific to the context in Uganda. Furthermore, other factors that could have influenced maternal healthcare utilization during the conflict, such the quality and availability of healthcare services, were not analysed due to limited scope of this study, as defined by the DHS variables available from the datasets. Finally, since many comparisons were made within the analyses, this may have increased the experiment-wise error rate of the results. However, when conducting exploratory analyses such as those in this study, it has previously been stated that correction for multiple tests is not always necessary^42^ .

## Conclusion

The findings in this study have shown that armed conflict can have a negative impact on maternal healthcare utilization, as previous studies have also suggested. However, certain aspects of maternal healthcare utilization, such as skilled assistance at birth in this study, show better results in conflict areas, likely as a result of humanitarian aid interventions to ensure the continuity of some healthcare services. In terms of the speed of post-conflict recovery, this study indicates that a 5-year period is too short to see any significant improvement in maternal health utilization rates.

The complex nature of armed conflict and the interplay of different factors such as conflict intensity, weakened health systems and services, and humanitarian aid, make it challenging to propose a set of concrete recommendations on how to improve maternal healthcare utilization in such settings. One needs to bear in mind that prevailing challenges at the societal, healthcare system and individual level are usually exacerbated by conflict, and humanitarian interventions are temporary in nature and are not meant to be sustainable in the long-term or replace previously existing services. Therefore, in addressing the problem of maternal healthcare utilization in conflict, strategies that target both improving the availability, accessibility and quality of existing services during conflict, as well as ‘building back better’ in terms of strengthening health systems post-conflict, need to be considered.

## Competing Interests

The authors have declared that no competing interests exist.

## Corresponding Author

Amrita Namasivayam: amrita.n10@gmail.com

## Data Availability

Data used for this project is owned by MEASURE DHS. Requests for this data can be made by registration and application via https://dhsprogram.com/data/Access-Instructions.cfm. The authors confirm that they accessed the data used for this study in this manner.

## References

[1] World Health Organization. Maternal Mortality 2016 [Available from: http://www.who.int/mediacentre/factsheets/fs348/en/

[2] World Health Organization Regional Office for Africa. Reducing maternal deaths: The challenge of the new millennium in the African region. 2002 [Available from: http://www.afro.who.int/drh/index.html.

[3] World Health Organization. Maternal mortality in 2005: estimates developed by WHO, UNICEF, UNFPA and the World Bank. 2007 [Available from: http://www.who.int/making_pregnancy_safer/topics/maternal_mortality/en/index.html.

[4] Sloan NL, Langer A, Hernandez B, Romero M, Winikoff B. The etiology of maternal mortality in developing countries: what do verbal autopsies tell us? Bull World Health Organ. 2001;79(9):805-10. PubMed PMID:11584727. 11584727 PMC2566647

[5] Campbell OM, Graham WJ. Strategies for reducing maternal mortality: getting on with what works. Lancet. 2006 Oct 7;368(9543):1284-99. PubMed PMID:17027735. 17027735 10.1016/S0140-6736(06)69381-1

[6] Bulatao RA, Ross JA. Which health services reduce maternal mortality? Evidence from ratings of maternal health services. Trop Med Int Health. 2003 Aug;8(8):710-21. PubMed PMID:12869092. 12869092 10.1046/j.1365-3156.2003.01083.x

[7] Ray AM, Salihu HM. The impact of maternal mortality interventions using traditional birth attendants and village midwives. J Obstet Gynaecol. 2004 Jan;24(1):5-11. PubMed PMID:14675972. 14675972 10.1080/01443610310001620206

[8] Khan KS, Wojdyla D, Say L, Gülmezoglu AM, Van Look PF. WHO analysis of causes of maternal death: a systematic review. Lancet. 2006 Apr 1;367(9516):1066-74. PubMed PMID:16581405. 16581405 10.1016/S0140-6736(06)68397-9

[9] McNamee P, Ternent L, Hussein J. Barriers in accessing maternal healthcare: evidence from low-and middle-income countries. Expert Rev Pharmacoecon Outcomes Res. 2009 Feb;9(1):41-8. PubMed PMID:19371178. 19371178 10.1586/14737167.9.1.41

[10] Simkhada B, Teijlingen ER, Porter M, Simkhada P. Factors affecting the utilization of antenatal care in developing countries: systematic review of the literature. J Adv Nurs. 2008 Feb;61(3):244-60. PubMed PMID:18197860. 18197860 10.1111/j.1365-2648.2007.04532.x

[11] Carroli G, Rooney C, Villar J. How effective is antenatal care in preventing maternal mortality and serious morbidity? An overview of the evidence. Paediatr Perinat Epidemiol. 2001 Jan;15 Suppl 1:1-42. PubMed PMID:11243499. 11243499 10.1046/j.1365-3016.2001.0150s1001.x

[12] Koblinsky MA, Campbell O, Heichelheim J. Organizing delivery care: what works for safe motherhood? Bull World Health Organ. 1999;77(5):399-406. PubMed PMID:10361757. 10361757 PMC2557673

[13] Graham, W.J., Bell, J., Bullough, C. H. W. (2001) Can skilled attendance at delivery reduce maternal mortality in developing countries? Studies Health Serv Organ Policy, 17: 97–130.

[14] Gabrysch S, Campbell OM. Still too far to walk: literature review of the determinants of delivery service use. BMC Pregnancy Childbirth. 2009 Aug 11;9:34. PubMed PMID:19671156. 19671156 10.1186/1471-2393-9-34 PMC2744662

[15] Bullough C, Meda N, Makowiecka K, Ronsmans C, Achadi EL, Hussein J. Current strategies for the reduction of maternal mortality. BJOG. 2005 Sep;112(9):1180-8. PubMed PMID:16101594. 16101594 10.1111/j.1471-0528.2005.00718.x

[16] Li XF, Fortney JA, Kotelchuck M, Glover LH. The postpartum period: the key to maternal mortality. Int J Gynaecol Obstet. 1996 Jul;54(1):1-10. PubMed PMID:8842811. 8842811 10.1016/0020-7292(96)02667-7

[17] Ronsmans C, Graham WJ. Maternal mortality: who, when, where, and why. Lancet. 2006 Sep 30;368(9542):1189-200. PubMed PMID:17011946. 17011946 10.1016/S0140-6736(06)69380-X

[18] The World Bank. Maternal mortality ratio (modeled estimate, per 100,000 live births) 2016 [Available from: http://data.worldbank.org/indicator/SH.STA.MMRT.

[19] World Health Organization. Maternal and Child Health: Uganda 2007 [Available from: http://www.who.int/pmnch/media/membernews/2011/ugandabackgroundpaper.pdf.

[20] Murray CJ, King G, Lopez AD, Tomijima N, Krug EG. Armed conflict as a public health problem. BMJ. 2002 Feb 9;324(7333):346-9. PubMed PMID:11834565. 11834565 10.1136/bmj.324.7333.346 PMC1122272

[21] Orach CG, De Brouwere V. Postemergency health services for refugee and host populations in Uganda, 1999-2002. Lancet. 2004 Aug 14-20;364(9434):611-2. PubMed PMID:15313362. 15313362 10.1016/S0140-6736(04)16854-2

[22] Gates, S., Hegre, H., Nygård, H., & Strand, H. Development Consequences of Armed Conflict. World Development. 2012, 40(9), 1713-1722.

[23] Ghobarah HA, Huth P, Russett B. The post-war public health effects of civil conflict. Soc Sci Med. 2004 Aug;59(4):869-84. PubMed PMID:15177842. 15177842 10.1016/j.socscimed.2003.11.043

[24] Plümper T, Neumayer E. The Unequal Burden of War: The Effect of Armed Conflict on the Gender Gap in Life Expectancy. International Organization. 2006;60(03).

[25] Urdal H, Che C. War and Gender Inequalities in Health: The Impact of Armed Conflict on Fertility and Maternal Mortality. International Interactions. 2013;39(4):489-510

[26] Chi PC, Bulage P, Urdal H, Sundby J. A qualitative study exploring the determinants of maternal health service uptake in post-conflict Burundi and Northern Uganda. BMC Pregnancy Childbirth. 2015 Feb 5;15:18. PubMed PMID:25652727. 25652727 10.1186/s12884-015-0449-8 PMC4327793

[27] US Department of State. The Lord's Resistance Army 2015 [Available from: http://www.state.gov/r/pa/prs/ps/2012/03/186734.htm.

[28] Swedish International Development Cooperation Agency. Uganda Strategic Conflict analysis 2006 [Available from: http://www.sida.se/contentassets/0c7a37d5972445e48f8dce4e7f8450b2/uganda-strategic-conflict-analysis_376.pdf.

[29] Armed Conflicts Reports. Uganda 2009 [Available from: https://www.justice.gov/sites/default/files/eoir/legacy/2014/02/25/Uganda.pdf.

[30] Chi PC, Bulage P, Urdal H, Sundby J. Barriers in the Delivery of Emergency Obstetric and Neonatal Care in Post-Conflict Africa: Qualitative Case Studies of Burundi and Northern Uganda. PLoS One. 2015;10(9):e0139120. PubMed PMID:26405800. 26405800 10.1371/journal.pone.0139120 PMC4583460

[31] Rutaremwa G, Wandera SO, Jhamba T, Akiror E, Kiconco A. Determinants of maternal health services utilization in Uganda. BMC Health Serv Res. 2015 Jul 17;15:271. PubMed PMID:26184765. 26184765 10.1186/s12913-015-0943-8 PMC4504353

[32] Magadi MA, Agwanda AO, Obare FO. A comparative analysis of the use of maternal health services between teenagers and older mothers in sub-Saharan Africa: evidence from Demographic and Health Surveys (DHS). Soc Sci Med. 2007 Mar;64(6):1311-25. PubMed PMID:17174017. 17174017 10.1016/j.socscimed.2006.11.004

[33] Kiwanuka SN, Ekirapa EK, Peterson S, Okui O, Rahman MH, Peters D, Pariyo GW. Access to and utilisation of health services for the poor in Uganda: a systematic review of available evidence. Trans R Soc Trop Med Hyg. 2008 Nov;102(11):1067-74. PubMed PMID:18565559. 18565559 10.1016/j.trstmh.2008.04.023

[34] Uganda Bureau of Statistics. Uganda Demographic and Health Survey 2011.

[35] Westoff CF. New estimates of unmet need and the demand for family planning 2006.

[36] Betsi NA, Koudou BG, Cissé G, Tschannen AB, Pignol AM, Ouattara Y, Madougou Z, Tanner M, Utzinger J. Effect of an armed conflict on human resources and health systems in Côte d'Ivoire: prevention of and care for people with HIV/AIDS. AIDS Care. 2006 May;18(4):356-65. PubMed PMID:16809113. 16809113 10.1080/09540120500200856

[37] Health care systems and conflict: a fragile state of affairs. PLoS Med. 2011 Jul;8(7):e1001065. PubMed PMID:21814498. 21814498 10.1371/journal.pmed.1001065 PMC3144195

[38] McGinn T, Austin J, Anfinson K, Amsalu R, Casey SE, Fadulalmula SI, Langston A, Lee-Jones L, Meyers J, Mubiru FK, Schlecht J, Sharer M, Yetter M. Family planning in conflict: results of cross-sectional baseline surveys in three African countries. Confl Health. 2011 Jul 13;5:11. PubMed PMID:21752241. 21752241 10.1186/1752-1505-5-11 PMC3162885

[39] Kruk ME, Freedman LP, Anglin GA, Waldman RJ. Rebuilding health systems to improve health and promote statebuilding in post-conflict countries: a theoretical framework and research agenda. Soc Sci Med. 2010 Jan;70(1):89-97. PubMed PMID:19850390. 19850390 10.1016/j.socscimed.2009.09.042

[40] ACLED. Armed Conflict Location and Event Data Project 2015 [Available from: https://www.acleddata.com/.

[41] Moss NE. Gender equity and socioeconomic inequality: a framework for the patterning of women's health. Soc Sci Med. 2002 Mar;54(5):649-61. PubMed PMID:11999484. 11999484 10.1016/s0277-9536(01)00115-0

[42] Drummond GB, Vowler SL. Type I: families, planning and errors. Clin Exp Pharmacol Physiol. 2012 Nov;39(11):897-900. PubMed PMID:23106692. 23106692 10.1111/1440-1681.12007

